# Differential Methylation Patterns in Apomictic vs. Sexual Genotypes of the Diplosporous Grass *Eragrostis curvula*

**DOI:** 10.3390/plants10050946

**Published:** 2021-05-10

**Authors:** Jose Carballo, Diego Zappacosta, Gianpiero Marconi, Jimena Gallardo, Marco Di Marsico, Cristian A. Gallo, Mario Caccamo, Emidio Albertini, Viviana Echenique

**Affiliations:** 1 Centro de Recursos Naturales Renovables de la Zona Semiárida (CERZOS–CCT–CONICET Bahía Blanca), Camino de la Carrindanga km 7, 8000 Bahía Blanca, Argentina; jcarballo@cerzos-conicet.gob.ar (J.C.); dczappa@criba.edu.ar (D.Z.); jgallardo@cerzos-conicet.gob.ar (J.G.); gallo.cristian.andres@gmail.com (C.A.G.); 2 Departamento de Agronomía, Universidad Nacional del Sur (UNS), San Andrés 800, 8000 Bahía Blanca, Argentina; 3 Dipartimento di Scienze Agrarie, Alimentari e Ambientali, Università degli Studi di Perugia, 06121 Perugia, Italy; gianpiero.marconi@unipg.it (G.M.); marco.dimarsico@studenti.unipg.it (M.D.M.); 4 NIAB, 93 Lawrence Weaver Road, Cambridge CB3 0LE, UK; Mario.Caccamo@niab.com

**Keywords:** apomixis, DNA methylation, diplospory, eragrostis, epigenetics

## Abstract

DNA methylation is an epigenetic mechanism by which a methyl group is added to a cytosine or an adenine. When located in a gene/regulatory sequence it may repress or de-repress genes, depending on the context and species. *Eragrostis curvula* is an apomictic grass in which facultative genotypes increases the frequency of sexual pistils triggered by epigenetic mechanisms. The aim of the present study was to look for correlations between the reproductive mode and specific methylated genes or genomic regions. To do so, plants with contrasting reproductive modes were investigated through MCSeEd (Methylation Context Sensitive Enzyme ddRad) showing higher levels of DNA methylation in apomictic genotypes. Moreover, an increased proportion of differentially methylated positions over the regulatory regions were observed, suggesting its possible role in regulation of gene expression. Interestingly, the methylation pathway was also found to be self-regulated since two of the main genes (*ROS1* and *ROS4*), involved in de-methylation, were found differentially methylated between genotypes with different reproductive behavior. Moreover, this work allowed us to detect several genes regulated by methylation that were previously found as differentially expressed in the comparisons between apomictic and sexual genotypes, linking DNA methylation to differences in reproductive mode.

## 1. Introduction

Epigenetics has become a key factor to understand mechanisms and pathways involved in regulation of the frequency, rate, or extent of gene expression without changing the DNA sequence. When epigenetics started to arise as a regulatory mechanism it was described as heritable changes in gene function that cannot be explained by changes in DNA sequence [1]. However, the modern definitions include long-term alterations in the transcriptional potential of a cell that are not necessarily heritable [2]. Particularly in plants, epigenetics is a mirror of the evolutionary history since they can exhibit remarkable phenotypic plasticity essential to colonize, grow, and reproduce in unpredictable terrestrial environments [3]. Epigenetic mechanisms are also key factors during the temporal and spatial fine-tune regulation of gene expression, thus enabling plants to survive and reproduce successfully in different environments [4]. The limits between epigenetic and genetic regulations are diffuse and the discussion of the scope of each one is still ongoing. However, there is a consensus that DNA methylation, histone modification, and RNA-mediated gene silencing are part of the epigenetic machinery. Histone modification and *de novo* DNA methylation are RNAi-dependent mechanisms; nevertheless, there is a continuous interaction between histone modifications and methylation [5].

DNA methylation in plants is a dynamic mechanism since methylation and demethylation processes are continuously occurring in relation to environment and development [6]. The addition of methyl groups to the DNA chain affects the chromatin structure and limits the transcription of the genetic information [6]. This mechanism is essential for plant homeostasis and development since its alteration produce abnormalities like failing in tomato ripening, decreasing the apical dominance and reducing the plant size in *Arabidopsis thaliana* and producing dark purple plants in *Zea mays* [7,8,9]. When demethylation mechanisms are activated, the DNA chain is again available to be transcribed and the normal development, if the disruption is not extremely severe, can be recovered [10].

DNA methylation in plants has been predominantly studied over the cytosine contexts where it takes place at the C5 carbon residue. The most common methylation target on cytosine’s residues is at CG sites, mediated by methyltransferases of the *MET1* family [11,12]. Non-CG methylation is also possible over the CHG and CHH contexts (H describe non-guanine residues). CHG and CHH methylation is mediated by *CMT3* and *CMT2* class of methyltransferases, respectively [13]. *De novo* methylation (i.e., newly methylated sites) appears to be primarily RNA-directed and requires the *DRM* (DNA dependent RNA methylation) gene family [14]. The demethylation process can be achieved actively through *ROS* [15], *DME* [16], and *DML* [17] glycosylases or passively by the failure of DNA methylation during DNA replication. The position of methylation marks over coding or non-coding regions of the genome is another fundamental factor for understanding the role of epigenetics in gene regulation. Gene body marks can repress or de-repress gene expression according to the context (CG, CHG, or CHH) and the species [3]. A common pattern in *A. thaliana* and *Oryza sativa* is the positive correlation between expression and methylation within the gene body in CG contexts. Repression usually occurs when the methylation marks (all contexts) are located in the TSS (transcript start site) and TTS (transcript termination site) boundaries [18]. This methylation pattern in the CG context is probably shared by all angiosperms since a study of 34 different species showed similar results [19]. Gene expression when CHG and CHH marks are present within the gene body seems to be species dependent [18], even though deeper studies are needed to better define this.

DNA methylation over the sixth position of the adenine ring (6mA) is one of the most abundant DNA modifications. Even though the evidence of this epigenetic mark has long been known [20], research works focusing on this topic has had a great impact in the last few years in part because the development of technologies able to detect the 6mA modifications [21,22,23]. The consequences of 6mA modifications over gene expression are not completely understood, however, in *O. sativa* and Arabidopsis it shows an additive effect to CG methylation since it represses gene expression when the methylation mark is around the TSS and promote the gene expression when the methylation mark is over the gene body [21,23]. 

Many technologies have been developed to detect both adenine and cytosine methylation marks over the genomes. Probably the bisulfite genomic sequencing analysis is one of the most used techniques to detect methylation in the cytosine contexts. It can assess the methylation status in all cytosines in a genome, however, the coverage needed to perform a high quality analysis (at least 5–10-fold) make the technique expensive and labor intensive, especially in studies with large genomes and multiple samples [24]. To overcome the DNA damage produced by bisulfite technique, the Enzymatic Methyl-seq (EM-seq) method was developed, which use lower DNA inputs and requires less coverage [25]. Other techniques are based on methylation sensitive endonucleases like methylation-sensitive digestion and sequencing (MRE-seq), the EpiRADseq variation of Double digest RADseq (ddRADseq) and the new method methylation content sensitive enzyme double-digest restriction-site-associated DNA (MCSeEd) [22]. The method is based on restriction-enzymes and allows the detection of changes in DNA methylation for the three cytosine (CG, CHG, and CHH) and the 6mA contexts, in genomic loci.

Apomixis is an asexual mode of reproduction where the progeny are genetically identical to the maternal plant [26]. Apomixis is scattered in multiple taxa; however, in the majority of the species three components are retained: apomeiosis (meiosis bypass), parthenogenesis (formation of an embryo without fertilization), and pseudogamy (fertilization of the polar nuclei without fertilization of the eggcell). Some apomictic species like *Hieracium* subgenus *Pilosella* can develop the endosperm without fertilization but this is not frequent in other plant species [27]. The regulation of apomixis remains unknown, however multiple hypothesis had been raised in the last years as product of several studies [28].

*Eragrostis curvula* (weeping lovegrass) is a grass originated in the south east of Africa, that exhibits different ploidy levels, from 2× to 8×. This species has become a model for diplosporous apomixis since the tetraploids have the whole range of reproductive modes (i.e., sexual, full, and facultative apomictic), lack of meiotic stages and the seed has the same embryo:endosperm ploidy ratio in both, sexual and apomictic genotypes [29]. This is due to its particular type of embryo sac development (Eragrostis-type) [30]. The reproductive mode of this grass was widely studied using different approaches like cyto-embryology [31], differential gene expression [32,33,34], mapping of the apomixis locus [35], and genome assembly [36]. Moreover, it was shown that internal and external stressful situations, like water stress, in vitro culture and intraspecific hybridization increase the percentage of sexual embryo sacs in facultative apomictic plants, evidencing the existence of some epigenetic control over the process [37,38]. More evidence of epigenetic mechanisms controlling apomixis were observed in this grass since transcriptional analyses and in situ hybridization of RdDM pathway genes revealed contrasting expression patterns for two genes (*EcAGO104* and *EcDMT102*) in apomictic vs. sexual plants [39]. Moreover, differentially expressed microRNA between sexual and apomictic genotypes were observed targeting transcripts encoding Squamosa Promoter-Binding-like (SPL) protein and a MADS-box transcription factor [40]. More recently we suggested that a DNA glycosylase *EcROS1*-like could be demethylating, thus de-repressing a gene or genes involved in sexuality pathways under water stress conditions [34].

More evidence of epigenetic mechanisms controlling apomixis comes from a study in *Paspalum simplex* and *P. notatum* where a reduction in the frequencies of parthenogenesis was observed when the plants were treated with the demethylating agent 5-azacytidine [41]. It was also observed that mutations in certain epigenetic regulators lead to the induction of apomictic elements in sexual plants like *A. thaliana* and *Z. mays* [42,43,44,45].

The aims of the present study were 1) to look for a correlation between reproductive modes (sexual, full, and facultative apomictic) and DNA methylation levels in *Eragrostis curvula* and 2) to depict the correlation between DNA methylation status and gene expression in apomictic vs. sexual plants [33]. To do so, a methylation content sensitive enzyme double-digest restriction-site-associated DNA (MCSeEd) technique was employed. 

## 2. Results

### 2.1. Differentially Methylated Positions (DMPs) and Differentially Methylated Regions (DMRs)

To infer if the reproductive behavior of *E. curvula* is due to differences in DNA methylation, DNA samples from panicles of plants with different reproductive modes were compared. In particular, the MCSeEd technique [22] was employed to identify differences in DNA methylation in facultative vs. sexual (FVS), full apomictic vs. sexual (AVS) and full apomictic vs. facultative (AVF) comparisons. To this end, genomic DNA was obtained from Don Walter (facultative apomict), Tanganyika (full apomict) and OTA-S (sexual) panicles. A total of 36 MCSeEd libraries were constructed by double restriction–ligations, with *Mse*I in combination with one of the four methylation-sensitive enzymes *Aci*I, *Pst*I, *Eco*T22I, and *Dpn*II for the CG, CHG, CHH, and 6mA contexts, respectively (Table S1).

An average of 4,662,929 150-bp-long reads per library was obtained (Table S1). Of these reads, 93.7% passed the quality controls and were aligned to the *E. curvula* Don Walter draft reference genome assembly and only reads that mapped at unique genomic positions were retained (Table S1). A total of 71,493,027 reads were mapped uniquely on the Don Walter genome assembly (44.9% of the total reads, with a minimum of 38.8% for *Eco*T22I, and a maximum of 48.2% for *Pst*I) and were classified as MCSeEd loci (Tables S1 and S2).

We were able to identify 1,233,059 loci containing cytosines (767,063 for CG, 194,206 for CHG, and 271,790 for CHH) and 1,279,616 loci containing adenines for the comparison FVS (Table S2) and 1,312,689 loci containing cytosines (838,621 for CG, 212,254 for CHG and 261,814 for CHH) and 1,273,700 loci containing adenines for the comparison between AVS genotypes (Table S2). Finally, in the AVF comparison 1,371,730 loci were identified containing cytosines (827,142 for CG, 236,351 for CHG and 308,237 for CHH) while 1,427,178 loci were found containing adenines (Table S2).

The MCSeEd loci were then normalized, filtered and analyzed with the MethylKit r package [46]. A total of 13,033 (for CG), 15,385 (for CHG), 18,954 (for CHH) and 72,350 (for 6mA) positions with significantly different methylation levels (Differentially Methylated Positions, DMPs) were identified in the FVS comparison (FDR < 0.05). The AVS comparison resulted in as many DMPs as 9909 for CG, 9598 for CHG, 12,978 for CHH, and 48,374 for 6mA (FDR < 0.05) whereas in the AVF 10,202, 14,026, 14,819, 61,487 DMPs were found for CG, CHG, CHH, and 6mA, respectively. The DMPs in the 6mA context were in all cases higher than in the cytosine ones, representing in all the comparisons around 60% of the total marks (Table S2).

A Principal Component (PC) analysis was performed to distinguish between samples based on DMPs. In the FVS comparison, the first latent component (PC1) accounted for 70.9%, 73.2%, 83.8%, and 69.1% of the total variance, for the CG, CHG, CHH, and 6mA contexts, respectively, clearly discriminating between facultative apomictic and sexual genotypes (Figure S1A). For the AVS comparison, the first latent component (PC1) accounted for 72.6%, 76.8%, 85.3%, and 72.5% of the total variance for the CG, CHG, CHH, and 6mA contexts, respectively, showing, also in this comparison, a clear discrimination between full apomictic and sexual genotypes (Figure S1B). Finally, when comparing AVF the PC1 variances were 66.3%, 73.9%, 82.3%, and 68.7% for the CG, CHG, CHH, and 6mA context, respectively (Figure S1C), showing in this case differences between full apomictic and facultative plants.

Moreover, when all the methylation differences between apomictic and sexual genotypes were considered 2.27-fold (CHH) to 4.45-fold (6mA) more DMPs were observed in facultative apomictic than in sexual plants (Table S2), and 1.67-fold (CG) to 1.93-fold (CHG) more DMPs in full apomictic than in sexual plants (Table S2). In the AVS comparison, similar methylation levels were found for the CHG (1.03-fold) and CHH (1.04-fold) contexts, whereas less methylations were found in the full apomictic for the CG (0.74-fold) and 6mA (0.63-fold) contexts. 

In addition to the DMPs, differentially methylated regions (DMRs) were identified as genomic regions where at least two DMPs with the same methylation pattern (de-methylated or methylated) were located within a specific window. A total of 8633 DMRs were scored in FVS (448 for CG, 459 for CHG, 383 for CHH and 7343 for 6mA context), and 4708 (362 for CG, 233 for CHG, 251 for CHH and 3862 for 6mA context, respectively) in AVS (Table S3) comparisons. In the AVF analysis a total of 5977 DMRs were found (307, 368, 232, and 5070 DMRs for the CG, CHG, CHH, and 6mA contexts, respectively).

**Figure 1:**
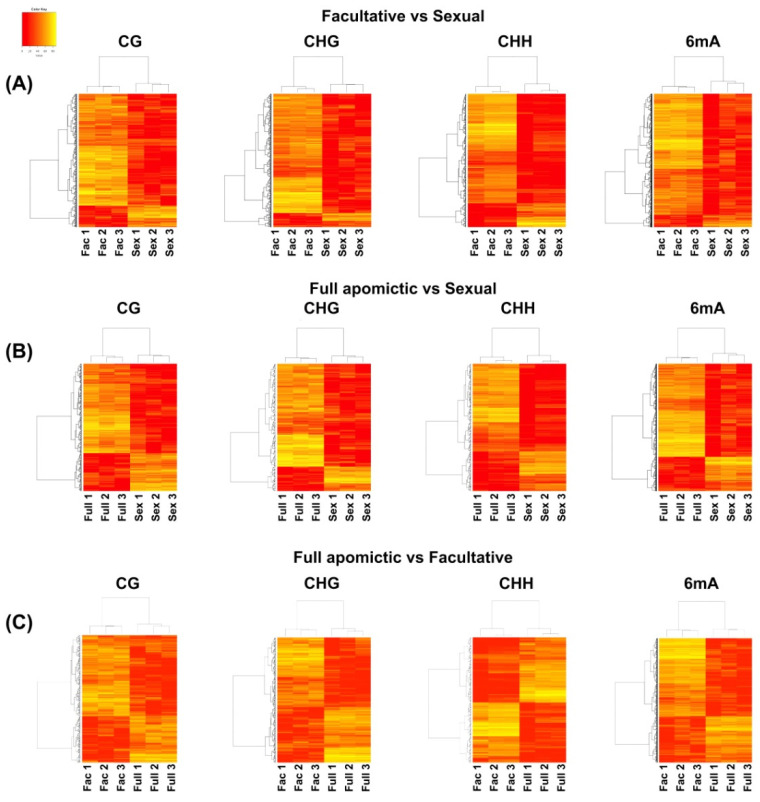
Relative methylation frequencies of the loci contained in each DMR identified from the comparison between the facultative apomictic or sexual (**A**), full apomictic or sexual (**B**) and facultative or full apomictic (**C**) plants. Sex: sexual plants, Fac: facultative apomictic plants, Full: full apomictic plants. Lower levels of relative methylation are indicated by more intense red color while higher levels of relative methylation are indicated by complete yellow.

The estimated relative methylation level of the loci belonging to each DMR were hierarchically clustered for all the analyses (Figure 1). The total number of DMRs for all the four contexts was higher in the FVS followed by AVF and AVS comparisons (Figure 1, Figures S2 and S3).

**Figure 2:**
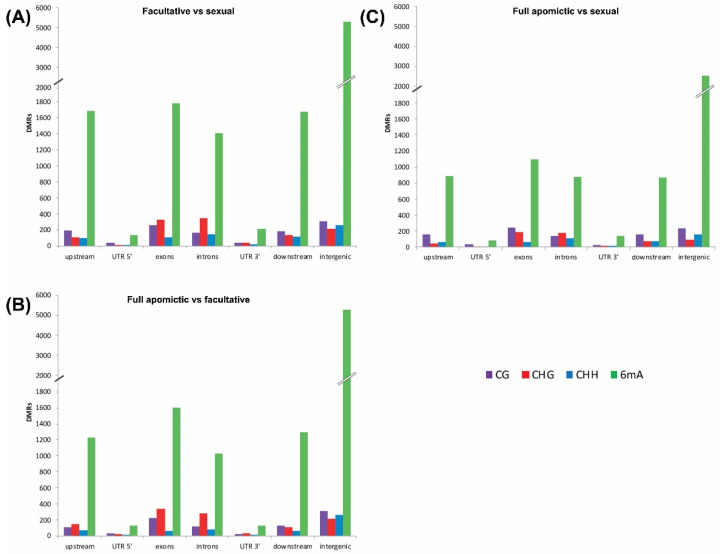
Number of DMRs overlapping genomic regions (X axis) in the Facultative vs. Sexual (**A**), Full Apomictic vs. Sexual (**B**) and Full Apomictic vs. Facultative (**C**) comparisons.

In order to assess the relationship between each methylated context and the genomic region being methylated (exons, introns, intergenic, etc.) the distribution of the DMRs across the Don Walter genome assembly was determined (Table S4; Figure 2). This analysis clearly showed that the CG, CHH, and 6mA contexts are the main methylation marks in intergenic regions. The CG context analysis showed also an important number of DMRs overlapping exons.

### 2.2. Identification of Differentially Methylated Genes (DMGs)

In order to understand how the methylation patterns of genic regions could impact on the expression of genes involved in the reproductive mode, the distribution of DMPs along DMRs was analyzed taking into account its position on coding and regulatory sequences (Extended Gene Bodies, EGB). In the comparison FVS a total of 395 upstream, 551 gene body, and 420 downstream regions were overlapped at least once by 1290 5mC-DMRs. The corresponding regions that overlapped with 7343 6mA-DMRs were 1664 upstream, 1857 gene body, and 1643 downstream (Table 1). In the AVS comparison a total of 270 upstream, 382 gene body, and 300 downstream regions were overlapped at least once by 846 5mC-DMRs, while the regions that overlapped with 3862 6mA-DMRs were 885, 1180, and 879 (Table 1). When comparing both apomictic genotypes (FVA) it was observed a total of 371 upstream, 479 gene body, and 356 downstream regions overlapping at least once by 907 5mC-DMRs and 1283 upstream, 1592 gene body, and 1172 downstream regions by 5070 6mA-DMRs (Table 1).

When comparing apomictic vs. sexual genotypes (FVS and AVS) in all methylation contexts it was possible to observe a higher number of methylated than de-methylated genes. However, when both apomictic genotypes were compared (Full apomictic vs. Facultative, AVF) the number of methylated and de-methylated regions was related to the context. In fact, a lower number of methylated positions was scored for the CG, CHH, and 6mA contexts, while an opposite pattern was observed for CHG.

**Figure 3:**
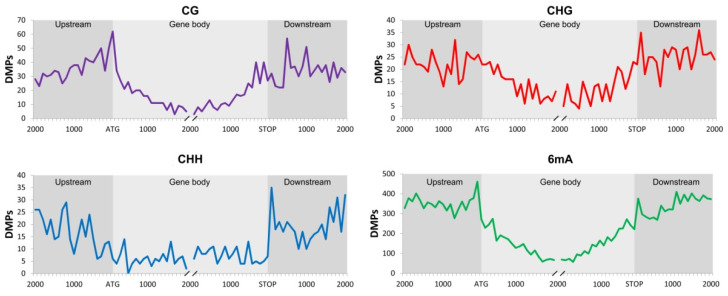
Differentially methylated positions along the EGBs for CG, CHG, CHH, and 6mA contexts. X axis correspond to 2000 bp before and after the ATG and STOP codon respectively.

To identify methylation patterns potentially affecting gene expression the distribution of DMPs belonging to DMRs along the three gene regions (upstream, gene body, and downstream) was analyzed (Figure 3).

Considering the aforementioned dataset, it was possible to observe more methylation marks over the regulatory regions for the 6mA context, meaning that probably genes are being regulated by methylation or de-methylation (repressed or active genes) in these regions. In the CG context, the number of methylation marks starts to increase from upstream to the ATG. An increase of methylations marks after the stop codon were observed in the CHG and CHH contexts. The methylation level in the gene body for all the contexts is lower than in the other regions. However, the number of DMPs in CHH and CHG contexts is more constant than in 6mA and CG. This could mean that regulation mediated by methylation in these contexts in the gene body could be more active than 6ma and CG context. 

**Figure 4:**
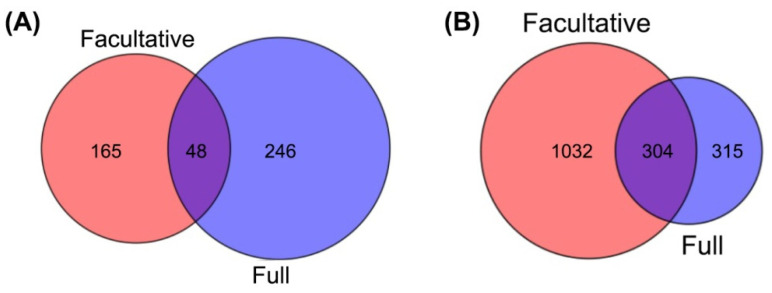
Venn diagram showing the de-methylated (**A**) and methylated (**B**) genes specific and shared between the full and facultative apomictic genotypes compared with the sexual one.

In order to identify genes involved in the apomictic development in *E. curvula*, methylated genes shared between the facultative and the fully apomictic genotypes (in the comparisons AVS and FVS) were analyzed. For this study only the genes methylated and de-methylated in the regulatory region and 400 bp after the start codon and before the stop codon were taken into account since it is believed that this methylation pattern is associated with a specific expression behavior (i.e., silenced when are methylated and active when de-methylated) [18]. This analysis allowed us to find 48 genes that were de-methylated in facultative and full apomictic genotypes when compared with sexual ones. A total of 246 and 165 genes showed to be de-methylated specifically in the fully apomictic and facultative apomictic cultivars, respectively, when compared with the sexual one (Figure 4A and Table S5). The genes that were found methylated in both facultative and full apomictic genotypes when compared to sexual ones accounted to be 304. The number of genes specifically methylated in the full apomictic respect to the sexual genotypes were 315 while those specifically methylated in the facultative apomict were 1032 (Figure 4B and Table S5). 

Twelve out of the 304 methylated genes and four out of the 48 de-methylated ones matched with F-box proteins at the Uniprot database.

**Figure 5:**
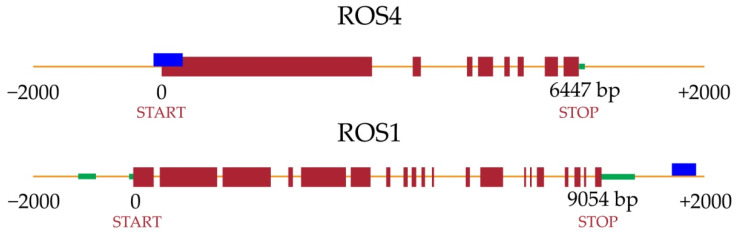
*ROS1* and *ROS4* genomic regions, showing 2000 bp before and after the start and stop codon, respectively. Blue rectangles represent DMRs, red rectangles exons and green rectangles UTR.

Interestingly, BTB/POZ and MATH domain-containing protein 1 (BPM1) and BTB/POZ and MATH domain-containing protein 2 (BPM2), FLOWERING LOCUS (FT) FAR1-RELATED SEQUENCE (FARS), Increased DNA Methylation 1 (IDM1/ROS4) and Repressor of Silencing 1 (ROS1) (Figure 5) were found within the 304 genes methylated in both facultative and obligate apomicts. These genes were previously related with the reproductive and/or methylation pathways.

ROS1 was found also within the 521 genes methylated in the AVF comparison (Figure 5, Table S5). The easiest interpretation is that even if the gene is repressed in all the apomictic genotypes, the level of repression depend on the degree of apomixis, i.e., being maximum in the full apomictic genotypes. Another interesting gene, found to be methylated is Cell Division Cycle 20.1 (*CDC 20.1*), which is indispensable for meiosis and male fertility. The AVF comparison resulted also in 903 de-methylated genes.

### 2.3. Gene Ontology Enrichment Analysis

**Figure 6:**
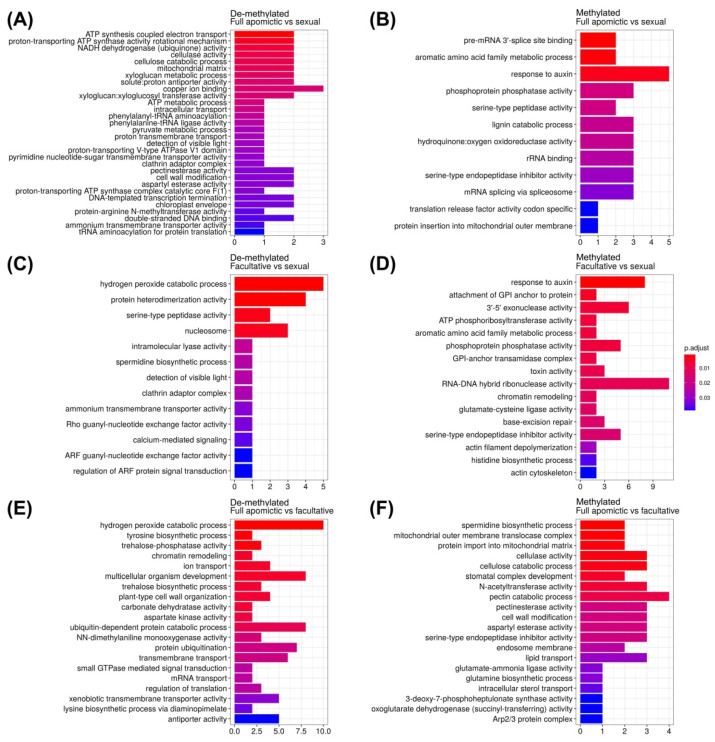
Differentially enriched GO terms considering de-methylated and methylated genes in the AVS (**A**,**B**), FVS (**C**,**D**), and AVF (**E**,**F**) comparisons.

We further investigated the gene ontology (GO) terms associated with the methylated and de-methylated genes over the regulatory regions, obtained from the three previous comparisons (Figure 3). The enrichment analysis was limited only to these regions since the correlation between the methylated positions in all the contexts and gene expression is high [18,19,21,23]. Interestingly, in the FVS and AVS analyses the term response to auxins was found enriched within methylated genes (Figure 6) as well as the terms aromatic amino acid family metabolic process, phosphoprotein phosphatase activity and serine-type endopeptidase inhibitor activity. Regarding the de-methylated genes, three terms were found in common between the FVS and AVS analyses: ammonium transmembrane transporter activity, detection of visible light and clathrin adaptor complex.

### 2.4. Differentially Methylated Genes and RNA-Seq Expression

One of the main advantages of *E. curvula* as a model diplosporous apomictic species is the coexistence of full apomictic and full sexual genotypes at tetraploid level. We previously used an RNA-seq approach to identify candidate genes for apomixis comparing the expression of genes in panicles of the sexual OTA-S and the full apomictic Tanganyika USDA cultivars [33]. The reads of this transcriptomes were mapped onto the Don Walter genome assembly to detect up and down-regulated genes for apomixis and compare them with the DMGs. Thus, the 1777 transcripts that were differentially expressed between apomictic and sexual genotypes (936 downregulated and 841 upregulated in the apomictic genotype) were compared with the DMGs, resulting in 44 unique genes that were both differentially ethylated and differentially expressed (Table S6).

The functional annotation performed using the Uniprot database [47] allowed to detect 27 matches out of the total 44 unique genes. Three *WAT1* genes were found in the 6mA context and downregulated in the RNA-seq study, two of them methylated and de-methylated in the gene body, respectively, and one methylated in the downstream region. In this comparison two methylated genes related to the ubiquitin pathway, an F-Box protein (6mA methylation in gene body, upregulated) and the ubiquitin-conjugating enzyme E2 23 (CHG methylation in gene body, downregulated) were also present.

## 3. Discussion

Our experimental design was aimed at finding a relationship between methylation changes over different genomic regions and the reproductive mode in *E. curvula*. In particular, we focused our attention on methylated and de-methylated genes and in their possible role in the regulation of the reproductive pathways. Previous findings in *E. curvula* allowed us to hypothesize that apomixis arise from the deregulation of the sexual development. However, this mechanism seems to be complex since several genes and pathways were found differentially expressed in apomictic vs. sexual comparisons [33,36,38].

The first analysis was aimed at inferring the number of Differentially Methylated Positions (DMPs) for the three comparisons (FVS, AVS, and AVF) in order to assess the methylation level in each genotype and the methylation contexts more affected by changes in apomictic and sexual plants. The analysis showed that in all comparisons DMPs for the 6mA context were higher than for the cytosine contexts, accounting for about 60% of the total marks. Similar frequencies were also found in previous studies in *Z. mays* with MCSeEd [22] and in *O. sativa* with mass spectrometry, DNA immunoprecipitation and single-molecule real-time sequencing [23], proving that, even when the cytosine context has been studied deeper, adenine seems to be more active in terms of changing their DNA methylation status.

Our results clearly show that the CG, CHH and 6mA contexts are the main methylation marks in intergenic regions even though an important number of CG-DMRs were found overlapping exons. The same methylation patterns were previously reported in *Z. mays* [22], in *A. thaliana* [21], and *O. sativa* [48]. Interestingly, CHG-DMRs were mainly located in exons and introns, suggesting that the regulation of gene expression by this context is exerted directly over the gene structure and not as for other contexts that act in intergenic regions. This CHG distribution pattern seems to be present also in other species like *Z. mays* [22,49] and *A. thaliana* [50].

In order to identify genes related to apomixis in *E. curvula*, genes with differentially methylated in regulatory regions and shared between the facultative and the full apomictic genotypes were evaluated (Figure 4). Interestingly, 12 out of the 304 shared methylated genes and four out of the 48 de-methylated genes were annotated as F-box proteins. Most of the F-box proteins participate in the recognition of target proteins degraded by ubiquitin protein ligase E3 and 26S proteasome-mediated. F-Box genes were found differentially expressed between apomictic and sexual nucellar tissues *of Boechera* [51]. Moreover, studies carried out in *E. curvula* [35], *Paspalum notatum* [52], *Hieracium praealtum* [53], and *Hypericum perforatum* [54] found genes belonging to the ubiquitin pathway as differential expressed between apomictic and sexual genotypes. This result shows that there might be a correlation between genes targeted by F-box proteins and reproductive behavior. Other genes of the ubiquitin pathway found methylated in apomictic genotypes when compared to sexual ones were *BTB/POZ* and *MATH domain-containing protein 1* (*BPM1*) and *BTB/POZ* and *MATH domain-containing protein 2* (*BPM2*), which serve as substrate adaptors to Cullin E3-ligases. In Arabidopsis, reduced expression of BPM1 resulted in an increased expression of ATHB6 protein and in a reduction of plant growth and fertility [55]. In the same species, *BPM2* can interact with members of the ethylene response factor/Apetala2 transcription factor family which are also involved in the reproductive pathway [56]. In *E. curvula* a *BPM2* gene was previously found both differentially methylated [37] and differentially expressed [34]. 

The GO analysis ran with the AVF data, showed that several methylated genes belong to the ubiquitin pathway, protein ubiquitination, and ubiquitin-dependent protein catabolic process terms. These findings suggest that methylation affect the expression of genes involved in the ubiquitin pathway which, in turn, regulate the abundance, activity and subcellular location of other plant proteins and this regulation cascade seems to be a plausible explanation for an intricate mechanism such as apomixis. However, more investigations are needed to shed light on the possible interconnections between apomixis and ubiquitination.

Other interesting genes found to be methylated in the apomictic plants were the Increased *DNA Methylation 1* (*IDM1*) and *Repressor of Silencing* 1 (*ROS1*). IDM1 gene is also known as *ROS4* because its mechanism of action is similar to that of *ROS1*, i.e., DNA demethylation [57]. The repression of *ROS1* and *ROS4* could decrease the expression of other genes since demethylation cannot be accomplished. *ROS4* identifies chromatin that contains CG methylation and low H3K4 and H3R2 methylations and catalyzes H3K18 and H3K23 acetylation, which then facilitates ROS1-mediated demethylation [58]. In *ROS1*, we found differences in methylation for the 6mA context thus suggesting a possible interconnection between 6mA and CG methylation, i.e., higher number of methylated CGs as consequence of the *ROS1* silencing due to the methylation of its 6mAs. This could partially explain the results obtained here (Table S7) and those previously reported by [21,23] where an additive effect to CG methylation mediated by 6mA was proposed. *ROS1* has also been related to an increased ratio of sexual embryo sacs in *E. curvula* [34]. However, the regulation of *ROS1* seems to depend on *ROS4* in the regulation cascade since *ROS4* generates histone acetylation marks (such as H3K18ac and H3K23ac) that are necessary for recruiting *ROS1* [57,58]. The consequence of the lack of function of *ROS1* and *ROS4* could be the silencing by methylation of a gene or genes necessary for a mechanism involved in the sexual pathway like meiosis or fertilization, hence, changing the cell fate, deregulating the sexual pathway, and possibly, giving rise to the apomictic development. When the DMPs were assessed in the AVF analysis, 903 genes were found de-methylated and 521 methylated. In the latter group, as well as in the AVS and FVS comparisons, the demethylating gene *ROS1* was also found, giving even more importance to this pathway in the regulation of plant reproduction. In *A. thaliana* the repression of *ROS1* was related to de-methylation at CG, CHG, and CHH contexts in the region upstream the start codon [59,60]. Our results show that the 6mA-DMRs are mainly located in the region downstream the stop codon, indicating that different methylation mechanisms could be governing the regulation of ROS1 in different plant species. De-methylations of some genes/genomic regions were previously associated with higher expression of ROS1 and to an increased ratio of sexual pistils in *E. curvula* under stress conditions [34,37]. The same study [37] showed that even when severely stressed, the full apomictic cultivar never produced sexual embryo sacs and this could be due to the higher level of methylation for certain genes like *ROS1* in full apomictic genotypes respect to facultative one. The flexibility of the mechanism, involving methylations and demethylations of key genes could explain in part the complexity of a facultative reproductive mode. Based on this assumption, the frequency of apomictic embryo sacs could be the result of a quantitative regulation that, under certain conditions, allows also the expression of sexuality.

Another interesting gene found within the 304 genes methylated in both full and facultative apomictic genotypes is represented by *FAR1-RELATED SEQUENCE (FARS)*. *FAR1* together with *FHY3* is involved in flowering time regulation by interacting with miR156-SPL whose targets are three *SQUAMOSA-PROMOTER BINDING PROTEINS-LIKE (SPL): SPL1, SPL2,* and *SPL3*. These genes inhibit key flowering regulatory genes, including *FRUITFUL* (*FUL*), *LEAFY (LFY)*, and *APETALA1 (AP1)* [61]. miR156 was previously found in *E. curvula* where it was actively involved in the silencing of three *SPL* transcripts [40]. Since *FAR1* is involved in the regulation of flowering and seed temporal and spatial development, and have a central role in reproductive pathways, its differential regulation by methylation/de-methylation could favor the arise of new components like apomeiosis, parthenogenesis or pseudogamy.

Within the 521 genes methylated in the AVF comparison, we found *Cell Division Cycle 20.1 (CDC 20.1),* which is fundamental for meiosis and male fertility. In *A. thaliana* the silencing of CDC 20.1 results in a delay of meiotic progression from diakinesis to anaphase I [62], making it a good candidate for further analysis. Another gene found within this group is *BABY BOOM 2* (*BBM2)*. The mutation of *BBM1*, *BBM2*, and *BBM3* cause embryo arrest and abortion, which can be fully rescued by male-transmitted *BBM1* or by the ectopic expression of *BBM1* [63]. Even when no differences were found in the AVS or FVS comparisons it was observed that this gene is differentially methylated between full and facultative apomictic plants. However, at the moment we cannot explain the biological meaning of such differences. 

The GO term response to auxin was found differentially enriched within the methylated genes (Figure 6). Auxins were reported as the triggers of seed development [64]. *YUCCA* genes, responsible for auxin biosynthesis in the ovule have a key function in the specification of embryo sac and egg cell development and their expression is related to *BBM1* [65,66]. In a previous work carried out in *E. curvula* [34] *YUCCA2* was found differentially expressed between control and water stressed plants. Genes found to be de-methylated both in the FVS and AVS comparisons mostly belonged to ammonium transmembrane transporter activity, detection of visible light and clathrin adaptor complex. Clathrins have been previously related to apomixis, specifically in the cell division process in Arabidopsis [67] and *Paspalum notatum* [50].

Finally, when we compared the DMGs and DEGs between the full apomictic and the sexual cultivars, we found that three *WAT1* genes showed different level of 6mA methylation that correlated with their downregulation in apomictic plants. *WAT1* genes are vacuolar auxin transporters, very similar in structure and function to *PIN* genes; both gene groups mediate intracellular auxin homoeostasis [68] and play a critical role in determining the directionality of auxin flows during embryonic and post-embryonic development [69].

## 4. Materials and Methods

### 4.1. Plant Material

Three tetraploid (2n = 4X = 40) genotypes of *Eragrostis curvula* with different reproductive modes were used: sexual (OTA-S PI574506, USDA, USA), full (Tanganyika PI234217, USDA, USA), and facultative (Don Walter, INTA, Argentina) apomicts. Plants were grown in 10 L pots in the greenhouse under controlled conditions with a photoperiod of 15 h light/9 h dark during the spring flowering period (Bahía Blanca, Argentina; 38°42′ S, 62°16′ W). All samples were taken from similar plants (same size, age, and growing conditions) to rule out environmental or growth effects that could influence the methylation status.

### 4.2. DNA Extraction

Genomic DNA was extracted from panicles according to Garbus et al. [33]. Briefly, fresh plant material was frozen and ground to a powder in liquid nitrogen using a TissueLyser II (Qiagen). For each sample, 100 mg of tissue was incubated at 65 °C in preheated extraction buffer containing 100 mM Tris HCl pH 8, 1.4 M NaCl, 20 mM, EDTA pH 8, 2% CTAB (*w*/*v*), and 0.5% (*v*/*v*) β-mercaptoethanol. Chloroform was subsequently added to reach a 2:1 ratio (buffer: chloroform) and the aqueous phase was collected after centrifugation. DNA was precipitated with one volume of isopropanol and washed with 70% (*v*/*v*) ethanol. The pellet was air-dried and resuspended in 50 µL of Milli-Q water containing 20 µg/mL RNase. All samples were quantified using a Qubit Fluorometer (Thermo-Fisher Scientific, Waltman, WA, USA) and DNA quality was determined based on its integrity in agarose gels.

### 4.3. Library Construction and Sequencing (DNA Methylation Analysis)

Libraries were constructed according to Marconi et al. [22]. Briefly, the enzyme chosen to infer CG, CHG, CHH, and 6mA methylation contexts were *Aci*I, *Pst*I, *Eco*T22I, and *Dpn*II, respectively, in combination with *Mse*I. For each library, 250 ng of DNA were double-digested with the four enzyme combinations following the protocol reported by Marconi et al. [22] and Di Marsico et al [70]. Libraries were then pooled (Table S8), purified using magnetic beads (Agencourt AMPure XP; Beckman Coulter, MA, USA), size selected by gel electrophoresis and purified using QIAquick Gel Extraction kits (Qiagen) for fragments in the range of 250 bp to 600 bp. Size-selected libraries were quantified using a fluorometer (Qubit; Life Technologies), and a normalized DNA amount (15 ng) was amplified with a primer that introduced an Illumina index (at the Y common adapter site) for demultiplexing. Samples were then amplified using uniquely indexed primers, pooled and subjected to PCR-enrichment as described by Marconi et al. [22]. The final library was Illumina-sequenced using 150 bp single-end chemistry. The raw reads were checked by quality analysis using the FastQC program (www.bioinformatics.babraham.aC.uk/projects/fastqc/, accessed on 1 April 2021) employing a pipeline developed by Novogene Company. Briefly, adapter sequences, duplicate sequences, reads containing N > 10% (where N represents the base cannot be determined), ambiguous and poor-quality reads (with a base count of Phred value <20), were removed using the TrimGalore program (https://www.bioinformatics.babraham.ac.uk/projects/trim_galore, accessed on 1 April 2020). Raw reads from the Illumina sequencing of the CG, CHG, CHH, and 6mA libraries were analyzed following the protocol and the pipeline described in Marconi et al. [22]. Reads were normalized and filtered discarding all of the sites with a co-logarithm of the variation coefficient value higher than −0.35 (Table S1). The relative methylation levels at each site were calculated following the procedure of Marconi et al. [22] and the DMPs (Differentially Methylated Positions) were called following the methylKit R package manual best practices [46]. The mapping of the DMPs was performed in the same best window and the DMRs were identified as was reported by Marconi et al [22]. Briefly, the first step was to maximize the number of DMRs in a set of adjacent windows in order to identify the best window length for each context. Therefore, a range of windows, from 100 to 2000 bp were tested. To do so, each potential window (i.e., 100 bp) was screened for DMPs that were significantly differentially methylated (false discovery rate, <0.05). The 5′-end of the window was therefore registered to start at the DMP position. Additional DMPs that were mapped within the re-positioned window (i.e., 100 bp) were included in the cluster, provided that the following conditions were met: (i) the direction of the methylation change agreed with the preceding DMP included in the cluster; and (ii) the DMPs to be included were called with a given significance threshold (false discovery rate, <0.05). After the inclusion of DMPs in the cluster the window start was registered to the position of the most 3’ end of the DMPs included, and the procedure was repeated as described. If no additional DMPs were identified based on the described conditions, the scanning procedure was restarted until a DMP was identified. The clusters that were composed of more than 2 DMPs were analyzed using logistic regression to identify and define the DMRs. Once the data for each window length was produced, the operator chose the best length, i.e., the one that maximized the number of DMRs per window. At this point, the script was re-started for each context using the adjacent window of the chosen length in bp (900 for CG, 1000 for CHG, 700 for CHH, and 400 for 6mA in the FVS, 1000 for CG, 900 for CHG, 700 for CHH, and 400 for 6mA in the AVS and 700 for CG, 900 for CHG, 900 for CHH, and 400 for 6mA in the AVF). Data are available under the SRA accession number PRJNA719488.

### 4.4. Differentially Methylated Genes (DMGs)

To analyze how the methylation patterns typical of genic regions could impact on the regulation of the reproductive mode DMP and DMR distributions were analyzed in relation to coding and regulatory genomic sequences (Extended Gene Bodies, EGB). The differentially methylated regions containing positions mapping on genes were analyzed by disaggregating the EGBs in gene body (5′ and 3′ UTR, introns, and exons), 2 kb downstream, and 2 kb upstream regions. The three regions in the four contexts (CG, CHG, CHH, and 6mA) were analyzed separately based on the correlation between gene expression and the methylated position. These regions were filtered if they have in the same region methylated and de-methylated positions in the same comparison regarding reproductive mode.

To assess the distribution of the methylated positions in the EGBs the whole set of genes differentially methylated in each context were plotted using a 100 bp window. Gene bodies were plotted at 2000 bp from the start and to the end of the gene models in order to have an optimal resolution of the distribution.

### 4.5. Gene Ontology Analysis

Gene ontology (GO) annotation was performed using the Interproscan 5 lookup service [71]. A differentially GO enrichment analysis was performed using the R package clusterProfiler [72] with methylated and de-methylated genes as datasets from full apomictic and facultative genotypes, and a *p*-value adjust cutoff of 0.05. The GO terms were plotted in a bar chart sorted by *p*-value and term count.

### 4.6. Differentially Expressed Genes 

A differential expression analysis was made using previously sequenced RNA-seq data from panicles of the full apomictic genotype Tanganyika USDA and the sexual genotype OTA-S [33]. First, the reads were mapped onto the Don Walter genome assembly using hisat2 software [73] and the reads that mapped on transcripts were extracted with GenomicFeatures package [74]. Then, a differential expression analysis was made using EdgeR package [75] in order to assess if the genes were up-regulated or down-regulated (i.e., genes highly expressed in the full apomictic cultivar Tanganyika USDA and low expressed in the sexual OTA-S). Finally, a cross comparison was performed in order to detect the genes differentially expressed in the RNA-seq experiment and differentially methylated in the MCSeEd analysis in order to detect genes regulated by methylation being overexpressed or repressed.

## 5. Conclusions

In this work we found that, in general, the apomictic genotypes exhibit higher DNA methylation levels than the sexual ones. The results showed here agree with previous findings, and therefore reinforce the hypothesis that the genes controlling the sexual pathways are present but repressed in apomictic plants. This repression is quantitative in facultative genotypes and total in full apomictic ones. The level of repression can be accomplished by different epigenetics mechanisms, like methylation. Moreover, a self-regulation of the methylation mechanism is acting since the demethylases ROS1 and ROS4 are being regulated by methylation and are differentially methylated between sexual and apomictic genotypes. Moreover, a strong connection between DNA methylation and key molecular pathways involved in reproduction like ubiquitination and auxins signaling was evidenced. Thus, taking into account all these results we can state that apomixis has a strong epigenetic component and methylation in certain pathways is crucial and affect the differential expression of genes between apomictic and sexual plants, and also between facultative versus full apomictic genotypes. More research in this direction is needed in order to better elucidate this intricate mechanism.

## Figures and Tables

**Table 1:** Number of methylated (Meth) and de-methylated (De-meth) genes at 2 kb upstream, gene body and 2 kb downstream regions in each context covered by a DMRs in the FVS, AVS, and AVF comparisons. DMRs represent the total number of regions per context.

		Upstream		Gene Body		Downstream	
		Meth	De-meth	Meth	De-meth	Meth	De-meth
Context	DMRs	**Facultative vs. Sexual**					
**CG**	448	160	29	139	46	141	31
**CHG**	459	98	16	214	29	117	22
**CHH**	383	76	16	110	13	89	20
**6mA**	7343	1473	191	1659	198	1484	159
		**Full Apomictic vs. Sexual**					
**CG**	362	108	55	94	63	109	44
**CHG**	233	35	10	113	25	53	18
**CHH**	251	36	26	66	21	55	21
**6mA**	3862	625	260	875	305	629	250
		**Full Apomictic vs. Facultative**					
**CG**	307	44	72	49	98	39	66
**CHG**	368	85	113	102	154	71	113
**CHH**	232	22	35	35	41	33	34
**6mA**	5070	462	821	543	1049	438	734

## Data Availability

SRA accession number PRJNA719488.

## Supplementary Materials

The following are available online at https://www.mdpi.com/article/10.3390/plants10050946/s1, Figure S1: DMPs Principal component analysis (PCA) for the FVS (A), AVS (B), and AVF (C) comparisons. Figure S2: Boxplot for each sample and context representing for the FVS (A), AVS (B), and AVF (C) comparisons. Figure S3: Boxplot with the samples collapsed for each genotype for the FVS (A), AVS (B), and AVF (C) comparisons. Table S1: Sequencing, trimming, mapping summary. Table S2: MCSeEd results. Table S3: DMPs and DMRs for the three comparisons. Table S4: Number of DMRs overlapping the different genomic regions. Table S5: Genes differentially methylated in regulatory regions. Table S6: genes in common between DMGs and differentially expressed genes for apomixis in RNA-seq. Table S7: Number of genes being methylated in more than one context. Table S8: Experimental design.
