# Supplementary figures and images for: Differential Methylation Patterns in Apomictic vs. Sexual Genotypes of the Diplosporous Grass Eragrostis curvula
Source: Plants (Basel). 2021 May 10;10(5):946. doi: 10.3390/plants10050946 (PMC8150776; doi:10.3390/plants10050946)

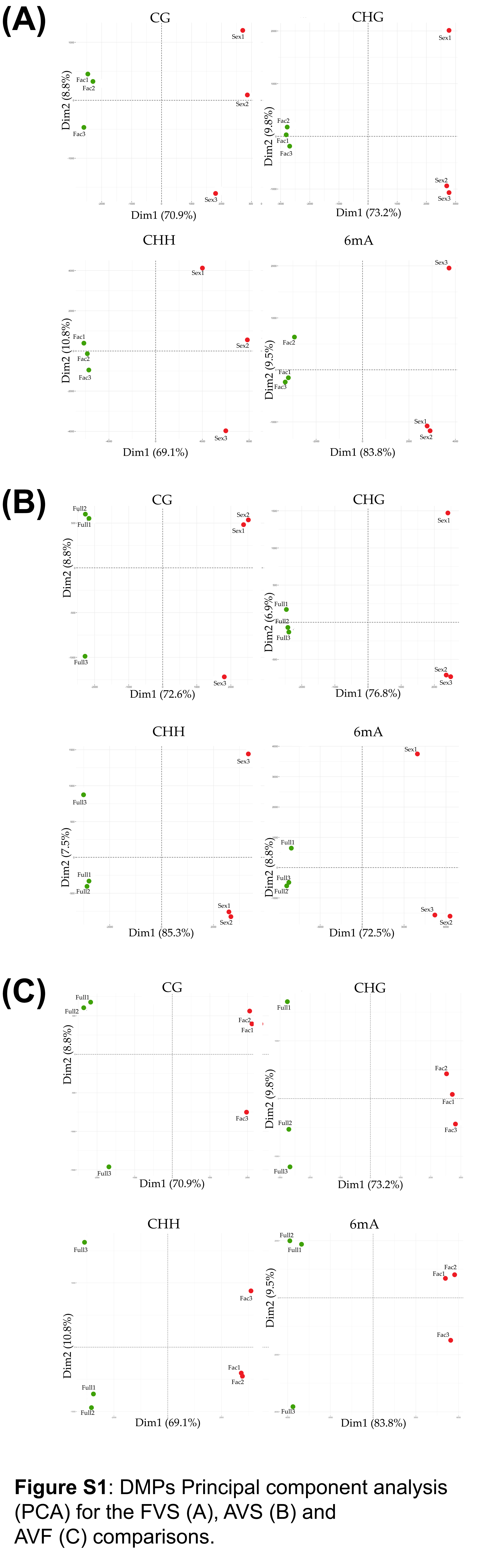

Supplement: Supplementary file 1 [file plants-10-00946-s001.zip › Figure S1.jpg]

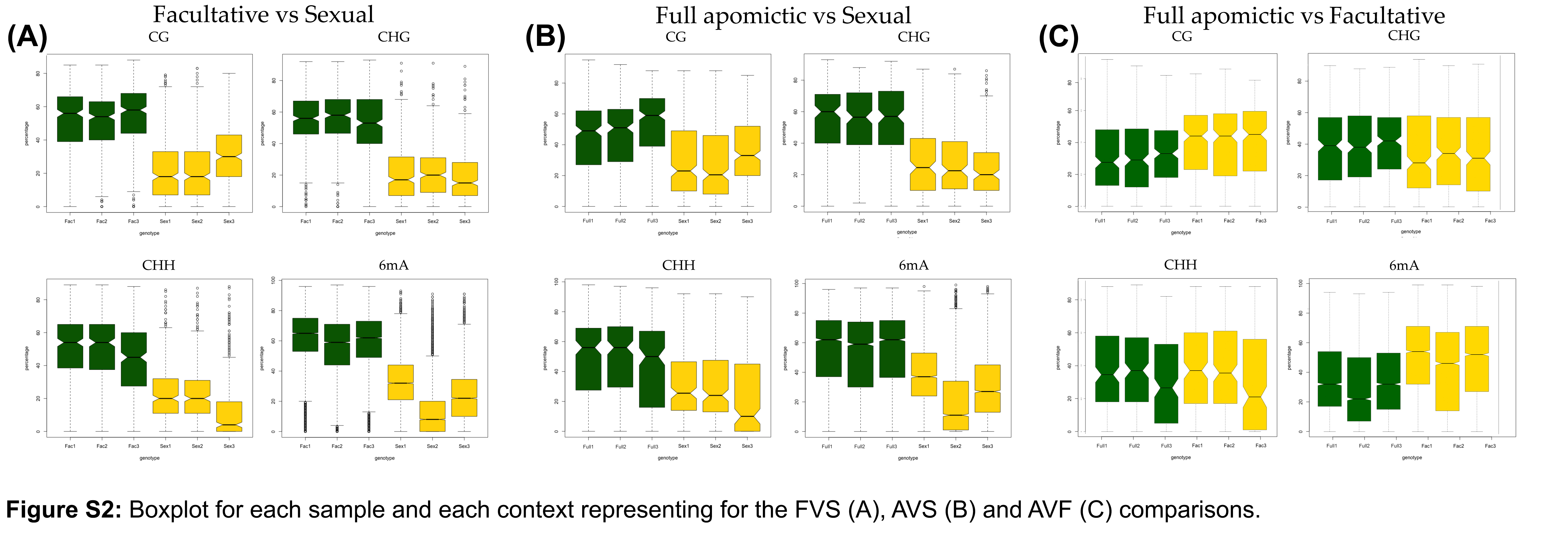

Supplement: Supplementary file 1 [file plants-10-00946-s001.zip › Figure S2.jpg]

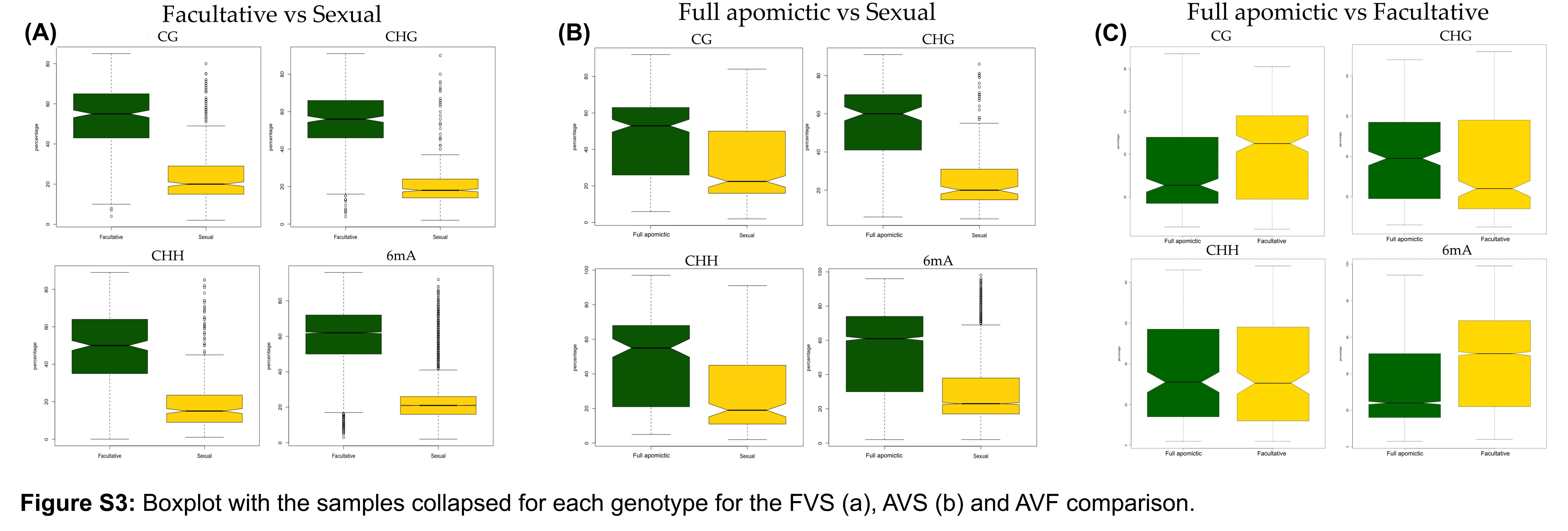

Supplement: Supplementary file 1 [file plants-10-00946-s001.zip › Figure S3.jpg]
